# Understanding health system reconstruction in conflict-affected states: a repeated cross-sectional study of healthcare coverage trends in Rwanda

**DOI:** 10.1186/s12913-026-14404-6

**Published:** 2026-03-20

**Authors:** Emily Ming Li Tan, Zangin Zeebari, Anneli Eriksson

**Affiliations:** 1 Department of Global Public Health, Karolinska Institutet, Stockholm, Sweden; 2 University of Oxford, Oxford, UK

**Keywords:** Health system, Conflict, Genocide, Reconstruction, Rwanda

## Abstract

**Background:**

Health systems are often a casualty of conflict, with widespread disruption caused by the loss of infrastructure, resources, and the workforce. The recovery of these systems, termed reconstruction or rehabilitation, is crucial to national recovery, but our understanding of this process remains limited. Rwanda experienced conflict in the form of genocide in 1994 which devastated the healthcare system. They have since made significant progress in health system development, providing a key case study to further our understanding of health system reconstruction. This paper aims to use a quantitative, health system-focused method to analyse the recovery of the Rwandan health system.

**Methods:**

This study uses a repeated cross-sectional design to examine trends in essential healthcare coverage in Rwanda, based on Demographic and Health Survey data between 1990 and 2019. Measures of maternal health provision were used as proxy markers for essential healthcare coverage. Trends were visualized using basis splines, and significant breaks were tested using post-estimation Wald tests. Piecewise beta linear regressions were used to clarify changes in trend.

**Results:**

Consistent trends across proxy markers demonstrate that healthcare coverage is decreased after conflict. Following a post-conflict lag of 3-10 years, healthcare coverage undergoes a marked increase in a rehabilitation phase present for all proxy markers. After 2011, healthcare coverage plateaus at a high level, providing evidence for a sustainable development phase in Rwanda’s post-conflict recovery. Primary care markers show increased coverage earlier than secondary care markers.

**Conclusion:**

The example of Rwanda suggests that key strategies for successful post-conflict reconstruction may include investment into primary care, early adoption of sustainable solutions, and strong internal co-ordination of reconstruction. Analysis of health system reconstruction should use health system-based approaches, and longer time frames of analysis. Further research should aim to create evidence-based resources that aid recently conflict-affected states.

**Supplementary Information:**

The online version contains supplementary material available at 10.1186/s12913-026-14404-6.

## Background

Fragile states affected by conflict are unlikely to meet the United Nation’s sustainable development goals by the intended target of 2030 [1, 2]. Conflict produces widespread effects on all aspects of sustainable development, which result in part from major disruption to the health system [3]. This disruption, which is often characterised by the loss of the ‘essential building blocks’ of a health system, is reflected in health indicators through a decrease in life expectancy and an increase in all-cause mortality (not just battle-related deaths) [4–6]. The recovery of a health system is therefore considered to be crucial to the recovery of a nation after conflict [7]. This process is commonly referred to as ‘health system rehabilitation’ or ‘reconstruction’. Whilst the term ‘resilience’ is often used in the literature, the ideas of strengthening and preservation that this encompasses can overlook the total devastation that results from conflict, since health systems, infrastructure, and human resources can become deliberate casualties [8, 9]. Accordingly, in this paper we will focus on reconstruction, defined as ‘the recovery and rebuilding of a health system after catastrophic shock’.

However, our understanding of these processes remains limited. Health system reconstruction is widely agreed to be challenging due to conflict and country-specific factors and the addition of international influences, all of which can hinder policy implementation and care provision [10]. Although the current body of literature continues to expand, much of the evidence is qualitative, focused on experiences of health system rebuilding, or is not systematic, analyzing only one aspect of a health system over a short timeframe using summary indicators (such as life expectancy at birth, or maternal mortality rate) [3, 11]. There have been two frameworks developed: the Waters framework published in 2007, and the Rutherford framework published in 2019. The Waters framework proposed a model of health system rehabilitation encompassing three synergistic and co-occurring processes: a response to immediate health needs, the restoration of essential services, and overall rehabilitation of the health system [10]. They concluded that these processes must account for policy-based inputs, such as the role of governance and non-governmental organizations, as well as material inputs, including health financing, infrastructure, medicines and technologies, and the healthcare workforce.

This framework was extended by Rutherford and colleagues in 2019, who sequenced the inputs and events that occur during post-conflict rehabilitation based on the comparison of three post-conflict case studies – Mozambique, Afghanistan, and Cambodia [3]. They determined four phases of recovery based on these examples: a response phase, a transitional phase, a rehabilitation phase, and an ‘ideal’ sustainable development phase. However, none of these three countries used as case studies had reached this final phase, and there is little evidence-based guidance for countries entering a sustainable development stage. Therefore, there is scope to use health system-based approaches on a range of case studies to improve success at the reconstruction of health systems after conflict.

One such case study is Rwanda, a country in eastern sub-Saharan Africa which experienced a catastrophic genocide in 1994 between the Hutu and Tutsi ethnic groups [12]. The country had experienced ongoing civil unrest since independence from Belgian colonisation in 1962, but the assassination of the Rwandan president and prominent Hutu Juvénal Habyarimana on the 6th of April 1994 sparked widespread mass killings. With the assassination attributed to the largely Tutsi Rwandan Patriotic Front, the Hutu government incited the military, general population, and paramilitary organisations to retaliate against their Tutsi neighbours. The subsequent genocide lasted 100 days, killing an estimated 800 000 to one million Tutsi and moderate Hutus, and creating 2 million refugees who fled to crowded and unsanitary camps in nearby countries [13]. This conflict devastated the healthcare system through loss of infrastructure and resources, and the death and desertion of the healthcare workforce [14, 15].

However, since this time, Rwanda has invested in significant health system recovery, through inputs at both the policy and resource levels in accordance with the Waters framework. In particular, the role of the Rwandan Ministry of Health (MOH) in coordinating the reconstruction effort is lauded for reducing fragmentation and increasing the sustainability of the policies implemented during the post-conflict period of 1995 and onwards [14, 16, 17]. Rwanda now has a health system which scored 49 on the World Health Organisation (WHO) Universal Healthcare Index in 2021, surpassing the African average of 44 [18].

Overall, the success of Rwanda’s post-conflict health system reconstruction provides a key case study for expanding our understanding of the reconstruction process. The aim of this paper is to extend our understanding of health system reconstruction, through assessment of the recovery of the Rwandan healthcare system after the 1994 genocide using a health system-focussed method. Visualizing changes in healthcare coverage during recovery has not yet been accomplished. We hypothesise that using a method of analysis which is focused on the health system (via essential service coverage) will provide a more detailed understanding of health system recovery than the use of summary indicators of national health and socioeconomics, and may suggest positive contributing factors which could be applied to other settings. In this paper, we propose a novel method of quantitative analysis of health system reconstruction, and use the case study of Rwanda to examine how this method may contribute to our understanding of system reconstruction after catastrophic conflict.

## Methods

### Study design

This study uses a repeated cross-sectional design to analyse changes in proxy markers of essential healthcare coverage in Rwanda from 1990 to 2019. Analysis of changing trends in healthcare coverage is done by compiling data from Demographic and Health Surveys (DHS) completed during the study period for the United States Agency for International Development. This time frame encompasses the pre-conflict period, the genocide, both Congo wars, and major healthcare reforms which have occurred in Rwanda since 1995, but excludes the recent Covid-19 pandemic, since this global event also affected health system capacity and resilience and therefore it is desirable to exclude the confounding effects of other types of catastrophic event. There are no data for the years 1993, 1994, and 1995 as no surveys covered these years. Data are aggregated by year, and the unit of analysis is the proportion of births within a given year for which a proxy marker was present. The use of aggregated data mitigates differences in sample size between years during the study period.

Essential healthcare coverage is represented by proxy markers of maternal healthcare service delivery. Research suggests that maternity care usage is shown not to significantly differ during conflict, indicating these services are representative of essential healthcare [19]. The signal functions of obstetric care, defined by the WHO as minimum standards for emergency obstetric care, also require a skilled healthcare attendant [20]. Maternal care markers have been used in the literature to study service delivery and health system resilience during conflict, indicating these markers can act as a measure of whether a health system can provide essential services [8, 19, 21, 22]. Additionally, data for these markers is often available when other data is not, such as during conflict. Essential healthcare coverage refers to the capacity of a health system to function based on essential service delivery. Essential services are defined as those which are commonly prioritized in post-conflict reconstruction, including basic curative care, care for communicable diseases, and maternal and child services [10].

### Data collection

Secondary data is taken from DHS surveys completed by local fieldworkers in Rwanda in the years 1992, 2000, 2005, 2010, 2014/15 and 2019/20. Data is from the women’s individual survey, collected from women aged 15–49, with complete records for only the most recent birth of each woman which occurred within the study year and four preceding years. The exposure variable is year of birth. The outcome variables are as follows:

- Proportion of births which received prenatal care from a skilled healthcare worker (primary care-associated).
- Proportion of births where the mother received a tetanus injection before birth (primary care-associated).
- Proportion of births which received birth assistance from a skilled healthcare worker (secondary care-associated).
- Proportion of births which took place in a healthcare facility (secondary care-associated).
- Proportion of births which occurred via Caesarean section, or C-section (secondary care-associated).
- Overall measure of essential healthcare coverage (an aggregated measure of the 5 individual proxy markers, where overall coverage is the mean number of markers per birth per year, as a proportion of total possible markers).

Data on the confounding variable, Gross Domestic Product (GDP) per capita, is taken from the World Bank database, which had complete data available for the study period [23].

### Statistical methods

For this analysis, a complete case approach was used, therefore records with missing data for any outcome variable were removed before analysis. Missing data was less than 1% for each variable. For each outcome variable, a 5-knot basis spline (b-spline) model was created to represent the data throughout the study period, with knots at 1990, 1997, 2004, 2011, and 2019. Visual analysis of b-splines was undertaken to identify obvious breaks in trend. Breaks were tested for significance using post-estimation Wald tests. Significant breaks were used to split the study period into segments, with a separate beta linear regression performed for each segment to clarify changes in trend. The chosen confounding variable was added to piecewise regression models if it returned significance in the regression models, or if its addition changed the marker co-efficient by greater than 1%. Consequently, it was included in the analysis of the individual proxy markers, but not the overall measure of essential healthcare coverage. Stata version 18.0 was used for this analysis, with a confidence level of 95% and significance level of 0.05.

## Results

Over the study period, 29,577 births were included in this study. The highest number of births surveyed was in 2004, with 1788 births, and the lowest number surveyed was in 2015, with 120 births. There was no clear trend in the change in number of births across the study period.

### Individual proxy markers of essential healthcare coverage

**Fig. 1:**
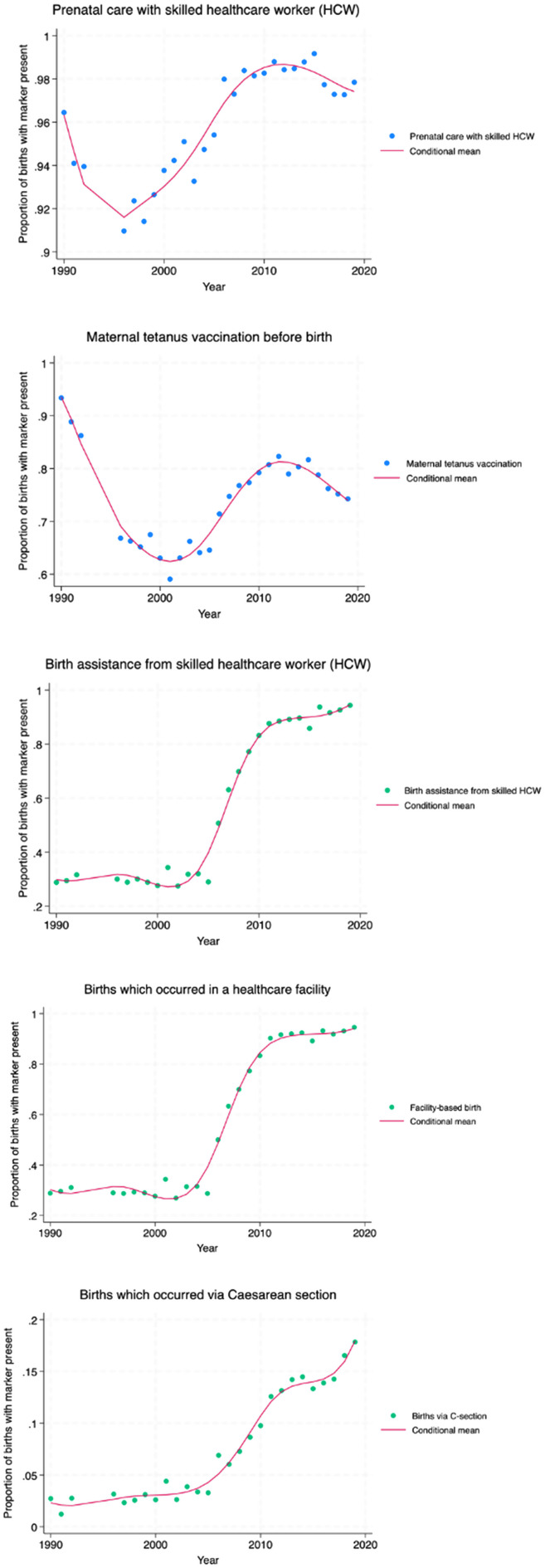
B-spline graphs for individual proxy markers of essential healthcare coverage

The b-spline graphs for the 5 individual proxy markers of essential healthcare demonstrate an overall increase in coverage for each marker from 1990 to 2019, with the exception of tetanus vaccination (Fig. 1). Similar patterns among primary care-associated (prenatal care from a skilled healthcare worker, maternal tetanus vaccination), and secondary care-associated markers (birth assistance from a skilled healthcare worker, facility-based birth, birth via C-section) were observed. Primary care-related markers show a decrease in coverage across conflict, followed by a period of marked increase which reaches an apex around 2011 before coverage begins to decrease again. Secondary care markers remain low for longer before undergoing a period of marked increase. After 2011, the trajectory of this increase reduces.

Significant breaks in trend which were visually identified were analysed using post-estimation Wald tests (Table 1). Individual markers associated with primary care have a significant break earlier than those related to secondary care, with prenatal care entering a period of marked increase in 1998, and tetanus vaccination in 2001, compared to secondary care markers which have the first significant break around 2005. All markers were found to have a significant break around 2011.

**Table 1:** Post-estimation Wald test results for significant breaks in trend for individual proxy markers

Outcome measure	Significant breaks	*P*-value
Prenatal care from skilled HCW	1998, 2011	< 0.001
Tetanus vaccination	2001, 2011	< 0.001
Birth assistance from skilled HCW	2005, 2011	< 0.001
Facility-based birth	2005, 2011	< 0.001
Birth via C-section	2005, 2011	< 0.001

Piecewise beta linear regressions were employed to clarify changes in trend. GDP per capita was included as a confounder for the individual proxy markers as it was significant for models of prenatal care in the first and second segments, and in most models caused changes in co-efficients of greater than 1%. After adjustment for GDP per capita, regression results generally agreed with the analysis of the b-spline graphs (Tables 2, 3 and 4). For the first segment (Table 2), primary care markers show.

significantly negative co-efficients, whereas the co-efficients for secondary care markers are close to 0, demonstrating negligible changes in coverage for this period (although only the co-efficient for C-sections was significant). This demonstrates an overall negative effect of conflict on healthcare coverage.

**Table 2:** Beta regression results of first segment for each individual proxy marker

Outcome measure	Time period	Co-efficient (β)	Pr > chi^2^ (model fit)	*P*-value of coefficient
Prenatal care from skilled healthcare worker	1990–1998	-0.048	< 0.001	< 0.001*
Tetanus vaccination	1990–2001	-0.152	0.037	**< 0.001**
Birth assistance from skilled healthcare worker	1990–2005	0.002	0.723	0.696
Facility-based birth	1990–2005	0.002	0.859	0.747
Birth via C-section	1990–2005	0.037	0.037	**0.005**

* Indicates that GDP was significant in the model

In the second segment (Table 3), all markers show a period of significant increase in coverage, which begins earlier for primary care markers. The magnitude of increase differs between markers, and depends on the starting level of coverage and subsequent overall change for each marker. For example, for prenatal care from a skilled healthcare worker, coverage remains above 90% for the whole study period, therefore during this segment coverage only increases by 9.6% of the previous year per year, although this change is significant (β = 0.096, *p* < 0.001). In contrast, facility-based birth, which begins the post-conflict period at 29% coverage, increases during this segment by 59.9% relative to the previous year per year, when adjusted for GDP per capita (β = 0.599, *p* = 0.002). As reflected by the Wald tests, this period ends around 2011 for all markers.

**Table 3:** Beta regression results of second segment for each individual proxy marker

Outcome measure	Time period	Co-efficient (β)	Pr > chi^2^ (model fit)	*P*-value of co-efficient
Prenatal care from skilled healthcare worker	1998–2011	0.096	< 0.001	< 0.001*
Tetanus vaccination	2001–2011	0.068	< 0.001	**0.036**
Birth assistance from skilled healthcare worker	2005–2011	0.529	< 0.001	**0.012**
Facility-based birth	2005–2011	0.599	< 0.001	**0.002**
Birth via C-section	2005–2011	0.312	< 0.001	**0.044**

* Indicates that GDP was significant in the model

In the third and final segment which occurs after 2011, there is a decrease in the trajectory of change seen across all markers (Table 4). For primary care markers, negative co-efficients representing a decrease in coverage are seen, although this is only significant for tetanus vaccination (β = -0.079, *p* = 0.021). For secondary care markers, coverage continues to increase but to a lesser degree than in the previous segment. The smaller co-efficient sizes and lack of significance for these markers indicate an end to the previous segment of increasing coverage, as increasing time is no longer significantly associated with increased coverage. All markers end the study period at a high level, with only tetanus vaccinations failing to exceed the pre-conflict level of coverage.

**Table 4:** – Beta regression results of third segment for each individual proxy marker

Outcome measure	Time period	Co-efficient (β)	Pr > chi^2^ (model fit)	*P*-value of co-efficient
Prenatal care from skilled healthcare worker	2011–2019	-0.241	0.033	0.051
Tetanus vaccination	2011–2019	-0.079	0.001	**0.021**
Birth assistance from skilled healthcare worker	2011–2019	0.137	0.014	0.074
Facility-based birth	2011–2019	0.013	0.064	0.827
Birth via C-section	2011–2019	0.002	0.001	0.922

### Overall measure of essential healthcare coverage

The same process of statistical analysis was employed to study the changes in an overall measure of essential healthcare coverage. This was an aggregate marker composed of the 5 individual proxy markers, where the mean number of proxy markers present per birth per year was used for the analysis. Across the study period, overall essential healthcare coverage increases from around 50% to around 75%. This just below the threshold of 80% of all markers for each birth, as not every birth should be a C-section.

**Fig. 2:**
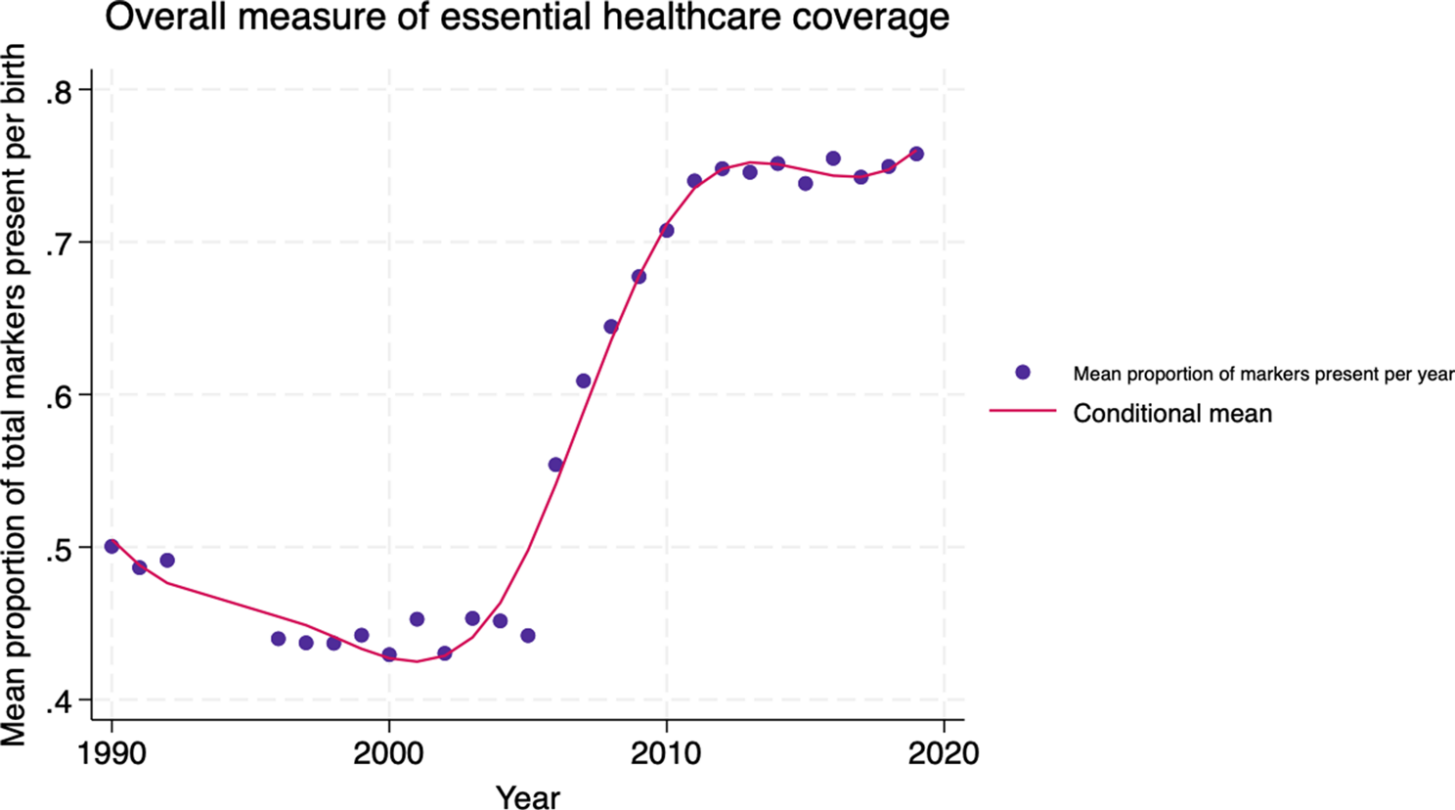
B-spline graph for overall measure of essential healthcare coverage

The trends seen in the b-spline analysis align with those seen in the b-splines for the individual markers (Fig. 2). A decrease is seen across conflict, as well as a period of marked increase which levels off around 2011. Figure 2 also reveals a period after conflict of sustained low coverage, producing a lag of around 7 years before essential coverage increases. Wald tests for visually identified significant breaks in trend confirmed breaks at 1998, 2005 and 2011. Iteration analysis was performed (1998 and 2005, 2005 and 2011, 1998 and 2011), with all permutations returning a significance level of *p* < 0.001.

The regression models for the overall measure of healthcare coverage follow the same trend seen in the b-spline analysis (Table 5). These models did not include GDP per capita as it did not return significance in any models, and co-efficients were not changed by more than 1%. Overall coverage significantly decreases across conflict (β = -0.035, *p* < 0.001). This is followed by a segment between 1998 and 2005 of little growth (β = 0.006, *p* = 0.222), representing a post-conflict lag in recovery of healthcare coverage. Between 2005 and 2011, a segment of marked increase reflects the results of the individual proxy markers, where increasing time leads to significant increases in coverage (β = 0.198, *p* < 0.001). After 2011, overall coverage plateaus at a high level, again reflecting the trends seen in the individual markers. Since the co-efficient for this segment was close to significance (β = 0.006, *p* = 0.081), some confidence can be assumed in this trend of static high coverage.

**Table 5:** Beta regression results for overall measure of essential healthcare coverage

Time period	Co-efficient (β)	Pr > chi^2^ (model fit)	*P*-value of co-efficient
1990–1998	-0.035	< 0.001	**< 0.001**
1998–2005	0.006	0.242	0.222
2005–2011	0.198	< 0.001	**< 0.001**
2011–2019	0.006	0.105	0.081

## Discussion

The aim of this paper was to extend our understanding of the health system reconstruction process, by analysing the case study of Rwanda using a novel quantitative health system-focused method. Markers of maternal health were used as proxy markers of essential healthcare provision, in accordance with the literature and the fact that maternal care usage does not significantly differ during conflict. By using these proxy markers to directly visualize changes in the function of the health system during the reconstructive period, we demonstrated the existence of a ‘post-conflict lag’, where essential coverage remained low after conflict. After this, we saw a defined period of marked increase, termed the ‘rehabilitation phase’ in accordance with the literature, until international target levels were met. Finally, we found evidence for a ‘sustainable development phase’, with sustained high levels of essential coverage. As conflict remains a pressing issue for public health, knowledge of these changes has several possible implications for research and implementation.

The defined period of marked increase in coverage, or the rehabilitation phase, is most visible from 2005 to 2011 in the overall measure of essential coverage. Knowledge of where this segment occurs in the overall trend of Rwanda’s post-conflict recovery could indicate inputs which were important to improving health system function. For example, many of the initiatives to augment primary care access and delivery occurred before 2005. The implementation of health posts for rural communities reduced walking time to healthcare from 95 min to 47, and the 1995 Community Health program introduced volunteer community health workers into local communities [24, 25]. This suggests that a focus on primary care in the immediate post-conflict phase could be foundational to the reconstruction of health systems.

This conclusion is further supported by the observation that increases in primary care-associated markers occur before increases in secondary care-associated markers. This may simply reflect a structured approach to health system reconstruction, as policies aimed at increasing access and quality of primary care were implemented early in the post-conflict period. Nevertheless, this approach is uncommon for many post-conflict settings, where donor investment is focused on improving secondary care within urban centres, neglecting basic primary care and care for rural areas [6]. In contrast, these results suggest that early investment into primary care and community access could be a useful approach that produces clear gains in the post-conflict reconstruction process.

A post conflict lag of between 3 and 10 years for primary and secondary care markers respectively, is also visible in the results of this study. Whilst the concept of a ‘response phase’ in the first year post-conflict was introduced in the Rutherford framework, this study found a much longer lag before coverage began to increase [3]. It may be that a post-conflict lag is most evident in health system-based approaches to analyzing post-conflict reconstruction. It is also important to remember that the Congo wars and refugee crisis took place during this time. Although not occurring on Rwandan soil, the indirect impact of these events on resources for health may have contributed to any lag effect. Nevertheless, the example of Rwanda suggests those working in reconstruction should not be discouraged by a lack of discernible results in the immediate post-conflict period, but focus on the implementation of sustainable initiatives. Further research may also determine ways through which to shorten this lag.

This study may also provide some evidence for the existence of a ‘sustainable development’ phase. Whilst this phase was first suggested by Rutherford and colleagues, they were not able to show an example of a country achieving this, perhaps due to a shorter analysis period of 12 years [3]. For this study, the 30-year period of analysis was necessary to see evidence for each distinct phase. Likely the period of analysis for post-conflict case studies should be much longer than is current practice. Analysis of factors which contributed to Rwanda’s attainment of the sustainable development phase may provide key insights for recently conflict-affected states.

Kruk and colleagues note that there is currently limited guidance for post-conflict states on when to transition from relief to development planning [6]. However, examples from Rwanda indicate that early initiatives for post-conflict reconstruction should consider sustainability and long-term development. For example, although the health posts were intended as a transitional solution, they have now been integrated into the health system with the creation of secondary ‘upgraded’ health posts which provide more extensive services [24]. These types of sustainable solutions are likely to be applicable to other settings, since they can be used to respond to a crisis, but are also able to be integrated into a recovering health system. Another example is the community-based health insurance system, or ‘*mutuelles de santé’*, which were piloted only 5 years after the conflict, but continue to promote health equity in the country [14, 26–28]. Development-minded solutions have likely contributed to Rwanda’s ability to achieve the sustainable development phase.

National ownership is also considered a critical mechanism through which sustainable reconstruction can be achieved. From the beginning of the post-conflict period in Rwanda, international aid was required to flow through the MOH into state-based programmes, which could then be sustained as international actors withdrew from the country [14, 17]. Settings such as South Sudan and Mozambique demonstrate how essential health services can be lost when international actors move their focus elsewhere, indicating the importance of state-based co-ordination of international aid to reduce fragmentation and overlapping vertical programmes [3, 29]. However, this may have been in part due to the stable governance of Rwanda from the early post-conflict period, a feature which is rare for a conflict-affected state, and therefore factors which have contributed positively to Rwanda’s rehabilitation may be difficult to apply to other settings.

Nevertheless, this work could provide much to consider for external aid agencies and multilateral international actors. The example of Rwanda contradicts the common assertion that external agencies should be the main driver of post-conflict health system reconstruction [3]. Whilst there is merit to the idea that external organizations take key roles in initiation and accountability, bypassing internal leadership during health system recovery leaves states with a poor long-term capacity for sustainable reconstruction and development, as seen with the case of Burundi [6, 17, 30]. Instead, a priority for multilateral organisations might be to provide guidance on the institution and maintenance of a strong body for internal co-ordination of reconstruction.

The results of this study should be considered in the context of the study limitations. It is likely that this method of analysis is oversimplified, focused mainly on service delivery and unable to account for the myriad complex factors affecting health systems in a conflict-affected setting [11]. Healthcare coverage is approximated from ‘essential services’, and may not account for services not prioritized during post-conflict reconstruction that are important for recovery, such as mental health services. Although the proportion of missing data was low, the use of complete case analysis may have introduced bias through record exclusion. The DHS survey samples may also not be representative of the overall population. The concept of the ‘post-conflict period’ is also somewhat arbitrary, as the end of overt hostilities does not preclude ongoing civil instability which may hinder development. Crucially, the role of GDP per capita was unable to be clarified in this study, and its relationship to healthcare coverage is likely more statistically complex that this method can account for. Lack of data also prevented the inclusion of other confounders, such as net financial assistance. Ultimately, refining this methodology through future research will likely provide more robust insights, although the consistency between the individual and overall measures of essential healthcare coverage implies the trends shown in the study are valid. Nonetheless, this research adds to the body of literature on health system reconstruction, and will provide a greater breadth of evidence to contribute to the creation of evidence-based guidance for conflict-affected states.

Lastly, it should be noted that since this research took place, United States Agency for International Development has been defunded, and data collection for the DHS programs has been suspended. The scale of data collection that DHS achieved has so far not been replicated elsewhere, and the loss of these data sets represents a huge loss for research into health systems reconstruction, and global health in general. Reliable data is needed to continue studying global health and health systems, and implementing changes which improve the health of those served by this work.

## Conclusion

Conflict remains a key issue for public health and policy. The use of a quantitative, health system-focused method to analyse the case study of Rwanda demonstrates that a closer understanding of health system changes during the post-conflict period can suggest strategies for improving our success at post-conflict reconstruction. Expanding similar health system-based methods, through analysis of a wider range of case studies, may produce further insights into the reconstruction process. Ultimately, greater investment into post-conflict reconstruction research should aim to produce evidence-based guidelines and resources for conflict-affected states.

## Supplementary Information

Below is the link to the electronic supplementary material.

Supplementary Material 1

## Data Availability

The data that support the findings of this study are available from the DHS survey program but restrictions apply to the availability of these data, which were approved for use for the current study, and so are not publicly available. Data are however available from the authors upon reasonable request and with permission of the DHS survey program.

## Abbreviations

B–spline: Basis spline
C–section: Caesarean section
DHS: Demographic and Health Surveys
GDP: Gross Domestic Product
MOH: Ministry of Health (in reference to the Rwandan Ministry of Health)
WHO: World Health Organisation
