# Supplementary material for: Understanding health system reconstruction in conflict-affected states: a repeated cross-sectional study of healthcare coverage trends in Rwanda
Source: BMC Health Serv Res. 2026 Mar 20;26:489. doi: 10.1186/s12913-026-14404-6 (PMC13064334; doi:10.1186/s12913-026-14404-6)
Supplement: Supplementary file 1 — Supplementary Material 1 [file 12913_2026_14404_MOESM1_ESM.docx]

**Supplementary methods**

**Outcome variables**

The variable for tetanus vaccination was coded by aggregating all records which had received between 1 and 7 tetanus vaccines, since the purpose of this study is to examine overall healthcare coverage rather than individual outcomes with prenatal vaccination. The variable for facility-based birth was created by aggregating all records where birth took place in a public or private healthcare facility, as determined by the DHS survey categories. Births which took place in a home, or where the answer was ‘don’t know’, were counted as ‘no’. The variable for the overall measure of coverage was an aggregated measure of the 5 individual outcome measures (prenatal care from a skilled healthcare worker, tetanus injection, birth assistance from a skilled healthcare worker, facility-based birth, as well as birth which occurred via C-section). The measure used was the mean number of markers per birth per year, as a proportion of total possible markers.

**Statistical methods**

Data cleaning

This study used complete case analysis, where records with missing data for any outcome variable were removed before analysis began. This method was used because the outcome variables are associated, and based on the survey design, records were likely to be complete. As a minor proportion of data was missing, and there was a low risk of bias, complete case analysis was used. Sample sizes after data cleaning can be seen in Table 1.

Statistical tests

Basis splines, also termed ‘b-splines’, are a statistical modelling method which are used to fit a predicted line around extremely non-linear data, using control points for curve segments known as knots. The use of b-splines was advantageous to allow a more accurate representation of trends in the data, reducing the influence of outliers. For these analyses, equally spaced knots were placed according to the protocol required for the Stata command. Models with 6, 5, 4 and 3 knots were tested using the Akaike Information Criterion (AIC), to determine a model which did not result in overfitting (as the AIC measure penalizes excess model complexity leading to overfitting). AIC test results can be seen in Table 2. Although the 3-knot model returned the lowest value for the marker of prenatal care from a skilled healthcare worker, overall, a 5-knot model was found to have the lowest value across the other proxy markers. Therefore, for comparability across models, a 5-knot model was used across the b-spline analyses for this project. Knots were as follows: 1990, 1997, 2004, 2011, and 2019.

Visual analysis of b-spline graphs was undertaken to find breaks in trend. Breaks were tested for significance using post-estimation Wald tests. These breaks were then used to split the study period into segments, to account for non-linear changes in trend. Piecewise beta linear regressions were performed for each different segment to analyse changes in coverage. The term ‘piecewise’ refers to the use of separate regressions around significant breaks which in combination account for the entire study period. Beta regressions were used because data was in the form of proportions, as beta regressions return co-efficients with a value between 0 and 1. For these regression models, time was considered a linear term.

The chosen confounding variable was added to piecewise regression models if it returned significance in the regression models, or if its addition changed the marker co-efficient by greater than 1%.

Analysis of individual healthcare proxy markers of essential healthcare coverage

B-spline analysis was used on each individual proxy marker of healthcare. Visual analysis of structural breaks was corroborated by Wald tests, and piecewise beta regressions were performed around significant structural breaks. GDP per capita was included in regression models for individual proxy markers because it was significant in models for prenatal care, and in most models caused changes in co-efficients of greater than 1%.

Analysis of overall measure of essential healthcare coverage

An overall measure of healthcare coverage in Rwanda was created using the 5 individual outcome variables: the proportion of births which received prenatal care from a skilled healthcare worker, the proportion of births where the mother received a tetanus injection, the proportion of births which received birth assistance from a skilled healthcare worker, the proportion of facility-based births, and the proportion of births which occurred via C-section. This assumes that women see either a doctor or a nurse for prenatal care and birth assistance, but are less likely to see both kinds of healthcare worker. The fact that not all births should take place via C-section is acknowledged, but the marker is included for greater breadth of analysis. For each birth, the number of markers present was calculated. The average number of markers per birth per year was then calculated as a proportion of total possible markers (in this case, 5).

B-spline analysis was performed using beta regressions, and piecewise beta regressions were performed after Wald testing of significant breaks identified through visual analysis. GDP per capita was not included in the regression models for this marker because it did not return significance in any models, and co-efficients were not changed by more than 1%.

Software

Statistical software used for this analysis is Stata version 18.0. A significance level of 95% was used across analyses, with a significance threshold of 0.05.

*Table 1 – Sample sizes after data cleaning*

| **Year** | **Number of births included in this study** |
| --- | --- |
| 1990 | 846 |
| 1991 | 1,068 |
| 1992 | 727 |
| 1996 | 476 |
| 1997 | 602 |
| 1998 | 861 |
| 1999 | 1,578 |
| 2000 | 1,269 |
| 2001 | 364 |
| 2002 | 877 |
| 2003 | 1,397 |
| 2004 | 1,788 |
| 2005 | 762 |
| 2006 | 696 |
| 2007 | 962 |
| 2008 | 1,429 |
| 2009 | 1,620 |
| 2010 | 1,506 |
| 2011 | 914 |
| 2012 | 1,209 |
| 2013 | 1,513 |
| 2014 | 1,657 |
| 2015 | 120 |
| 2016 | 929 |
| 2017 | 1,290 |
| 2018 | 1,536 |
| 2019 | 1,581 |

*Table 2 – AIC test results for knot-number specification in b-spline models*

|  | **6 knots** | **5 knots** | **4 knots** | **3 knots** |
| --- | --- | --- | --- | --- |
| **Prenatal care from skilled healthcare worker** | -183.025 | -183.910 | -180.467 | -186.001 |
| **Maternal tetanus vaccination** | -133.474 | -134.510 | -127.558 | -133.390 |
| **Birth assistance from skilled healthcare worker** | -97.434 | -102.537 | -82.574 | -95.392 |
| **Facility-based birth** | -103.779 | -105.996 | -96.172 | -92.778 |
| **Birth via C-section** | -168.949 | -170.414 | -160.656 | -169.734 |
| **Overall measure of essential healthcare coverage** | -44.461 | -48.983 | -28.786 | -36.206 |
